# Comparison of Physiological and Perceptional Responses to 5-m Forward, Forward-Backward, and Lateral Shuttle Running

**DOI:** 10.3389/fphys.2021.780699

**Published:** 2022-02-17

**Authors:** Chong Gao, Xiaolu Wang, Guochao Zhang, Li Huang, Mengyuan Han, Bo Li, George P. Nassis, Yongming Li

**Affiliations:** ^1^School of Physical Education and Sport Training, Shanghai University of Sport, Shanghai, China; ^2^College of Physical Education and Health Sciences, Longyan University, Longyan, China; ^3^School of Physical Education and Sport Science, Fujian Normal University, Fuzhou, China; ^4^Physical Education Department, College of Education, United Arab Emirates University, Al Ain, United Arab Emirates; ^5^Department of Sports Science and Clinical Biomechanics, SDU Sport and Health Sciences Cluster (SHSC), Faculty of Health Sciences, University of Southern Denmark, Odense, Denmark; ^6^China Institute of Sport Science, Beijing, China

**Keywords:** shuttle runs, change of direction, running modes, physiological demands, oxygen consumption

## Abstract

**Purpose:**

The aim of this study was to investigate the physiological and perceptional responses to forward, forward-backward, and lateral shuttle running.

**Methods:**

Twenty-four eligible male subjects performed a maximal oxygen uptake (VO_2max_) test and three directional modes (i.e., forward, forward-backward, and lateral) of 5-m shuttle running at the speed of 6 km⋅h^–1^ for 5 min on separate days. Heart rate (HR) and oxygen uptake (VO_2_) were continuously measured during the whole tests. Rating of perceived exertion (RPE) was inquired and recorded immediately after the test. Capillary blood samples were collected from the earlobe during the recovery to determine the peak value of blood lactate concentration ([La^–^]_peak_).

**Results:**

Running directional mode had significant effects on HR (*F* = 72.761, *P* < 0.001, η^2^_*p*_ = 0.760), %HR_max_ (*F* = 75.896, *P* < 0.001, η^2^_*p*_ = 0.767), VO_2_ (*F* = 110.320, *P* < 0.001, η^2^_*p*_ = 0.827), %VO_2max_ (*F* = 108.883, *P* < 0.001, η^2^_*p*_ = 0.826), [La^–^]_peak_ (*F* = 55.529, *P* < 0.001, η^2^_*p*_ = 0.707), and RPE (*F* = 26.268, *P* < 0.001, η^2^_*p*_ = 0.533). All variables were significantly different between conditions (*P* ≤ 0.026), with the variables highest in lateral shuttle running and lowest in forward shuttle running. The effect sizes indicated large magnitude in the differences of all variables between conditions (ES = 0.86–2.83, large) except the difference of RPE between forward and forward-backward shuttle running (ES = 0.62, moderate).

**Conclusion:**

These findings suggest that the physiological and perceptional responses in shuttle running at the same speed depend on the directional mode, with the responses highest in lateral shuttle running, and lowest in forward shuttle running.

## Introduction

Shuttle running, in which acceleration, deceleration, and 180°change of direction (COD) are performed successively at a given distance, is often used in either sport training (Zadro et al., 2011) or fitness evaluations (Léger et al., 1988; Stewart et al., 2014). It can be not only performed at submaximal speed (Hatamoto et al., 2014; Stevens et al., 2015) but also be applied in high-intensity interval training (Bravo et al., 2008; Haydar et al., 2011; Zadro et al., 2011). Due to the speed fluctuation and directional change (Dellal et al., 2010), shuttle running may produce additional physical load when compared with constant straight-forward running at the same average speed (Osgnach et al., 2010; Zago et al., 2018). It has been widely proved that the inclusion of COD during running could elicit greater heart rate (HR), oxygen uptake (VO_2_), rating of perceived exertion (RPE), and blood lactate concentration ([La^–^]) than linear running for both submaximal (Buchheit et al., 2011) and high intensity (Dellal et al., 2010).

Shuttle running can be manipulated by the distance between each turning, mean speed, and directional mode. Previous studies focused primarily on the distance and speed of shuttle running. The distance usually used in shuttle running was 5 m, 10 m, or 20 m. For the same mean speed of shuttle runs, shorter distance indicates higher COD frequency and larger speed fluctuation. The peak value of blood lactate concentration ([La^–^]_peak_) could reach 3.8 ± 2.6 mmol⋅L^–1^ (Buchheit et al., 2011) and 9.78 ± 3.05 mmol⋅L^–1^ (Zago et al., 2018) after 20-m and 5-m shuttle running with 75% maximal aerobic speed for 5 min. Hatamoto et al. (2014) explored the effect of COD frequency on the energy cost of shuttle running at different speeds. The results indicated that VO_2_, HR, and RPE rose almost linearly as the COD frequency increased from 13 to 18 times per minute at different velocities (3, 4, 5, 6, and 7 km⋅h^–1^). Ashton and Twist (2015) demonstrated that more directional changes during intermittent shuttle running increased the physiological load on female athletes. With regard to the same distance shuttle runs, there was a consensus that the HR, RPE, and VO_2_ per unit time was significantly higher at greater speed (Buchheit et al., 2011; Hatamoto et al., 2013) while the results of VO_2_ per unit distance in different researches didn’t come to an agreement. Buchheit et al. (2011) reported that there was no significant difference in VO_2_ per meter between different speeds (45, 60, and 75% of maximal aerobic speed), whereas Stevens et al. (2015) and Zago et al. (2018) claimed that it would be significantly higher when the speed increased. However, researchers mainly focused on forward running, which has been widely studied in either the physiological demands or the acute biomechanical determinants (Ishii et al., 2011; Zamparo et al., 2015; Zago et al., 2019, 2021). Although forward running has received much attention, other directional modes of shuttle running have been less concerned.

Running can be briefly divided into forward, backward, lateral based on the direction, and these running modes exist in daily activities and sports. Since backward running and lateral running are not habitual exercise for humans, they are expected to induce higher physiological and perceptional responses than forward locomotion. A few studies compared the differences of physiological and perceptional responses between backward and forward running on the treadmill (Flynn et al., 1994; Masumoto et al., 2009), yet lateral running was seldom incorporated in the experiment design. Indeed, moving backwards or laterally is often integrated with COD in many sports, such as basketball, soccer, handball, badminton, and tennis (Williford et al., 1998; Maurus et al., 2019). Nevertheless, until now, there has been no published research on the comparison of physiological and perceptional responses to shuttle running with different directional modes. Moreover, knowledge about the physiological demands of exercise is useful to assess training/exercise load and to formulate training plans, exercise prescription, fatigue prevention strategies, and nutrition management (Zago et al., 2018).

Therefore, the present study was designed to investigate the physiological and perceptional responses to shuttle running with three directional modes (i.e., forward, forward-backward, and lateral running). We hypothesized that: (1) directional mode had significant effects on the physiological and perceptional responses to shuttle running, and (2) the physiological and perceptional responses to shuttle running were highest in lateral shuttle running, and lowest in forward shuttling running.

## Materials and Methods

### Participants

Twenty-eight male college students volunteered to participate in this study. All participants had more than 2 years (up to 10 years) soccer training experience and spent more than 6 h on physical exercises every week in the last 4 weeks. A Physical Activity Readiness Questionnaire (PAR-Q) was used to exclude subjects with cardiovascular or musculoskeletal problems. Four of them were not included in the final analysis because of data loss or injury problems during the whole procedures. Twenty-four eligible subjects had the following characteristics (mean ± SD): age = 19.6 ± 0.8 years, height = 175.4 ± 6.2 cm, weight = 67.1 ± 5.9 kg, VO_2max_ = 60.3 ± 4.1 ml⋅kg^–1^⋅min^–1^. All the subjects included were in good health, not taking medications known to influence metabolic or cognitive functions.

### Experimental Design

The experiment consisted of a maximal oxygen uptake (VO_2max_) test and three shuttle running tests, including forward shuttle running, forward-backward shuttle running, and lateral shuttle running. HR, VO_2_, [La^–^]_peak_, and RPE were recorded to reflect the physiological and perceptional responses of the exercises. Subjects were also instructed to refrain from high intensity and strenuous exercises during the last 24 h ahead of each test, to standardize their food and drink intake (no caffeine), as well as to have a regular sleep on test days. The purpose, procedures and risks of this study were explained to the participants both verbally and in written format. Prior to the formal tests, all participants provided informed consent document after understanding the whole tests and got familiar with the test protocols and experimental instruments. This study was approved by the Research Ethical Committee of Shanghai University of Sport (approval number: 102772020RT085).

### Procedures

#### Maximal Oxygen Uptake Test

The subject’s VO_2max_ was determined with an incremental test on a treadmill (Saturn^®^ 300/100 r, H/P/Cosmos, Nussdorf-Traunstein, Germany) in a laboratory with the ambient temperature ranged from 20.3 to 23.5°C and the atmosphere pressure ranged from 100.8 to 102.7 kPa. The speed was increased by 1 km⋅h^–1^ every minute from an initial intensity of 8 km⋅h^–1^ and 1% gradient (Bravo et al., 2008; Kramer et al., 2018). HR and respiratory gas exchange were under real-time supervision with a HR monitor (T31, Polar-Electro, Kempele, Finland) and a portable gas analyzer (MetaMax 3B, Cortex, Leipzig, Germany). The gas analyzer was calibrated with ambient air (assumed concentration of 20.94% O_2_ and 0.03% CO_2_) and a standard gas of known composition (O_2_: 15.00%, CO_2_: 5.00%) for gas calibration, as well as a 3-L syringe for volume calibration according to the manufacturer’s instructions before the test. The subject’s rating of perceived exertion was assessed using Borg 6–20 scale (Borg, 1998) when the test was stopped. A capillary blood sample was collected from subject’s earlobe at the beginning of the 1st, 3rd, 5th, 7th, and 10th minute during the recovery. [La^–^] was analyzed with EKF lactate analyzer (Biosen C_line, EKF Diagnostic, Magdeburg, Germany). The test was immediately terminated if the subject waved hand to the researcher that indicated exhaustion. Other standards for termination were: (1) touching the handrail, (2) unsteady gait, and (3) failed to keep himself in the first half of the treadmill. Heart rate was recorded simultaneously with the respiratory data, which was collected continuously (mobile mean value of three consecutive breaths) using the breath-by-breath method. HR_max_ was the maximal value obtained during the test. VO_2max_ was defined as the maximum moving average value in 30-s window (Stevens et al., 2015) and expressed in milliliters of oxygen per kilogram per minute (ml⋅kg^–1^⋅min^–1^). To ensure the VO_2max_ was reached, the participant should meet at least 3 of the 5 following criteria (Howley et al., 1995; Midgley et al., 2007; Hatamoto et al., 2014; Ázara et al., 2017): (1) a VO_2_ plateau despite increasing speed (ΔVO_2_ between two consecutive stages < 2.1 ml⋅kg^−1^⋅min^−1^), (2) HR ≥ 95% individual age-predicted maximal HR (HR_max_ = 220 –age), (3) RER (respiratory exchange ratio, the ratio between the expired CO_2_ and the amount of O_2_ being consumed) ≥ 1.10, (4) RPE ≥ 18, and (5) [La^–^] ≥ 8 mmol⋅L^−1^. If not, the subject should take another VO_2max_ test with same procedures after at least 48 h.

#### Shuttle Running Tests

Forward, forward-backward, and lateral shuttle running tests were conducted by each subject in a random order on separate days. For each trial, participants performed a 180°COD after covering 5 m at a mean speed of 6 km⋅h^–1^ and repeated this continuously for 5 min in order to reach a metabolic steady state (Ciprandi et al., 2018; Zago et al., 2018). The selection of 6 km⋅h^−1^ was because participants could hardly complete the lateral shuttle running at higher speeds for 5 min, as we have investigated in pilot study. Conditions took place at the same time (± 0.5 h) of day. Each participant was required to wear the same running shoes and similar suits for the tests. All protocols were conducted on the same flat synthetic rubber indoor course where ambient temperature ranged from 19 to 23°C and atmosphere pressure ranged from 101.7 to 103.2 kPa during the shuttle running tests.

Prior to starting each test, participant performed a 10-min warm-up including 5-min jogging and 5-min dynamic stretches as dictated by the researcher. Then, instruments which had been calibrated according to the manufacturers’ instructions were assembled and checked. During all the tests, running pace was governed by a metronomic device. The audio signal beeped every 3 s and indicated the moment that the participants needed to change direction and the supporting foot should pass over the turning line. Right foot and left foot were alternatively used to change direction in order to avoid unilateral fatigue (Ciprandi et al., 2018; Zago et al., 2018). Each participant chose his own preferred stride length and stride frequency because it has been demonstrated that naturally selected stride length-frequency combination resulted in the lowest oxygen consumption (Hogberg, 1952; Cavanagh and Williams, 1982; Mercer et al., 2008). In forward shuttle running, subjects were instructed to change direction with a sidestep technique. Body was accelerated toward the direction opposite of the push-off leg (Schot et al., 1995; Cochrane et al., 2007; Potter et al., 2014) and the pivoting foot should land perpendicularly to the running direction (Zago et al., 2018). Since participants faced the same direction without any body rotation throughout the forward-backward or lateral shuttle running, there was no special technical requirement in these two conditions. A researcher supervised the whole tests and provided oral instruction if the participant failed to pass over the turning line or follow the pace.

Heart rate and respiratory gas exchange were continuously measured with a HR monitor (T31, Polar-Electro, Kempele, Finland) and a portable gas analyzer (MetaMax 3B, Cortex, Leipzig, Germany). HR was determined as the mean of HR in the last 2 min (Hatamoto et al., 2013). The average value of VO_2_ (ml⋅kg^–1^⋅min^–1^) in the last 2 min (representing a steady state) was calculated to represent the VO_2_ of this condition (Buchheit et al., 2011; Hatamoto et al., 2013; Zamparo et al., 2014, 2015, 2016). Relative HR_max_ and relative VO_2max_ were represented as %HR_max_ and %VO_2max_, which meant the ratio (in percentage form) of HR to individual HR_max_ and VO_2_ to individual VO_2max_, respectively. When the test was completed, the participant’s RPE [according to 6–20 Borg’s scale, Borg (1998)] was immediately inquired and recorded. During the recovery in sitting position, a capillary blood sample (10 μL) was collected from the participant’s earlobe in the 1st, 3rd, 5th, and 7th minute after each test (Li et al., 2018) and [La^–^]_peak_ was used to analyze in this study. Since the data were compared within subject and we assumed that the resting metabolic rate fluctuated within a very narrow range during the test days, the pre-test values were not considered (Buglione and di Prampero, 2013; Zamparo et al., 2014).

### Statistics

Data analyses were conducted with an academic statistics software (SPSS 25.0, IBM, Armonk, New York, NY, United States). Descriptive statistics were presented as mean ± SD within 95% confidence interval (95% CI) for all variables. The normality of each dependent variable was examined by Shapiro-Wilk test. When the normality distribution was rejected, the data were transformed by taking the natural logarithm to allow statistical comparisons that assume a normal distribution (Buchheit et al., 2011). Back-transformed values were presented in the text. The effect of running modes on the variables investigated in this study was assessed using a one-way analysis of variance (ANOVA) for repeated measures. Greenhouse–Geisser correction was performed if the sphericity assumption was violated. When significant effects were found, Bonferroni *post hoc* tests were applied to determine the differences between running modes. Effect sizes (Cohen’s *d*) of *post hoc* tests were calculated for the magnitude of differences. Threshold values were ≤ 0.20 (trivial), > 0.20–0.50 (small), > 0.50–0.80 (moderate), and > 0.80 (large), respectively. The level of statistical significance was set at *p* < 0.05.

## Results

Physiological and perceptional responses to three modes of shuttle running are shown in Table 1. Statistical results revealed that running mode had significant effects on HR (*F* = 72.761, *P* < 0.001, η^2^_*p*_ = 0.760), %HR_max_ (*F* = 75.896, *P* < 0.001, η^2^_*p*_ = 0.767), VO_2_ (*F* = 110.320, *P* < 0.001, η^2^_*p*_ = 0.827), %VO_2max_ (*F* = 108.883, *P* < 0.001, η^2^_*p*_ = 0.826), [La^–^]_peak_ (*F* = 55.529, *P* < 0.001, η^2^_*p*_ = 0.707), and RPE (*F* = 26.268, *P* < 0.001, η^2^_*p*_ = 0.533).

**TABLE 1:** Physiological and perceptional responses to three modes of 5-m shuttle running at 6 km⋅h^–1^.

	HR (bpm)	%HR_max_ (%)	VO_2_ (ml⋅kg^–1^⋅min^–1^)	%VO_2max_ (%)	[La^–^]_peak_ (mmol⋅L^–1^)	RPE
Forward	136.1 ± 13.0	69.8 ± 5.56	34.4 ± 2.47	57.3 ± 5.29	1.73 ± 0.53	8.83 ± 1.49
	*(109.1, 163.1)*	*(58.2, 81.4)*	*(29.3, 39.6)*	*(46.3, 68.3)*	*(0.62, 2.83)*	*(5.72, 12.0)*
Forward- backward	147.8 ± 14.7*	75.8 ± 6.19*	38.7 ± 3.69*	64.4 ± 7.04*	2.66 ± 1.00*	9.75 ± 1.59^*^
	*(117.1, 178.5)*	*(62.9, 88.7)*	*(31.0, 46.4)*	*(49.7, 79.2)*	*(0.58, 4.74)*	*(6.42, 13.1)*
Lateral	158.7 ± 16.7^* †^	81.4 ± 7.32^* †^	43.3 ± 3.89^* †^	72.1 ± 7.62^* †^	4.73 ± 2.25^* †^	11.3 ± 1.33^* †^
	*(123.9, 193.5)*	*(66.1, 96.7)*	*(35.2, 51.4)*	*(56.2, 88.0)*	*(0.03, 9.43)*	*(8.48, 14.0)*

*Values are mean ± SD.*

*Italics in brackets are the 95% confidence interval (95% CI) for each parameter.*

*HR, heart rate; HR_max_, maximal heart rate; %HR_max_, the ratio of HR to HR_max_ in percentage; VO_2_, oxygen uptake; VO_2max_, maximal oxygen uptake; %VO_2max_, the ratio of VO_2_ to VO_2max_ in percentage; [La^–^]_peak_, the peak value of blood lactate concentration; RPE, rating of perceived exertion.*

*Statistically significant difference vs. forward mode (p < 0.05).

*^†^Statistically significant difference vs. forward-backward mode (p < 0.05).*

**FIGURE 1:**
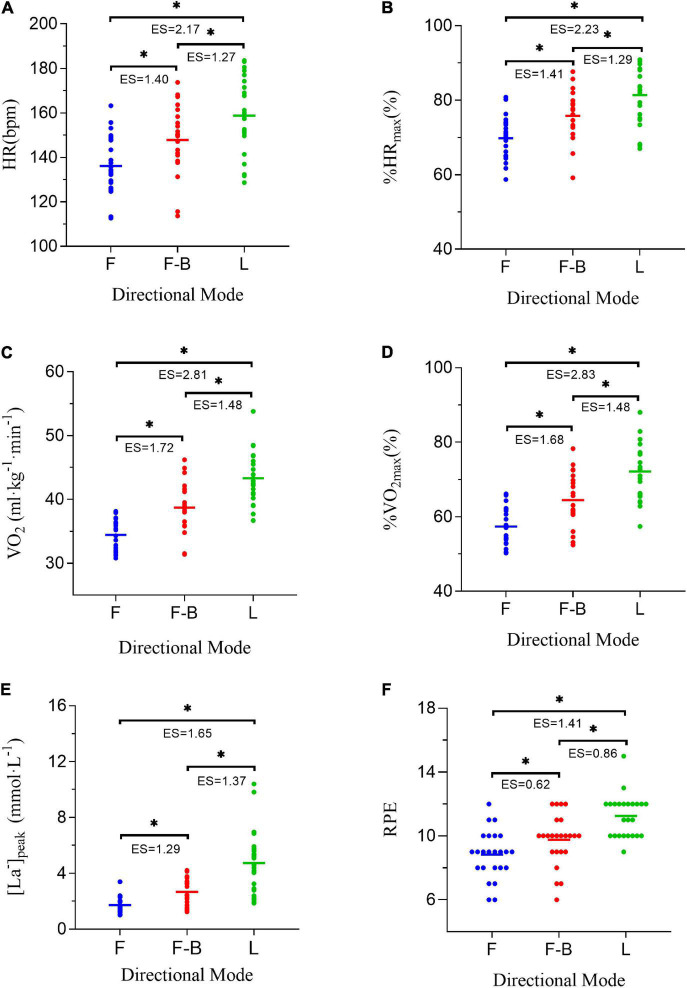
Physiological and perceptional responses to three modes of shuttle running: HR **(A)**, %HR_max_ **(B)**, VO_2_ **(C)**, %VO_2max_ **(D)**, [La^–^]_peak_ **(E)**, RPE **(F)**. HR, heart rate; HRmax, maximal heart rate; %HR_max_, the ratio of HR to HR_max_ in percentage; VO_2_, oxygen uptake; VO_2max_, maximal oxygen uptake; %VO_2max_, the ratio of VO_2_ to VO_2max_ in percentage; [La^–^]_peak_, the peak value of blood lactate concentration; RPE, rating of perceived exertion; F, forward shuttle running, F-B, forward-backward shuttle running; L, lateral shuttle running; ES, effect size. *Statistically significant difference (*p* < 0.05).

Bonferroni *post hoc* tests showed that all variables investigated in this study were significantly different between conditions. HR, %HR_max_, VO_2_, %VO_2max_, [La^–^]_peak_, and RPE in forward-backward shuttle running were higher than in forward exercise. Lateral shuttle running yielded the highest responses. The effect sizes indicated large magnitude in the differences of all variables between conditions except the difference of RPE between forward and forward-backward shuttle running. The individual physiological and perceptional responses to the same condition varied much between subjects (see Figure 1).

## Discussion

The present study evaluated the HR, %HR_max_, VO_2_, %VO_2max_, [La^–^]_peak_, and RPE of 5-m forward, forward-backward, and lateral shuttle running at the speed of 6 km⋅h^–1^. To our knowledge, it is probably the first study to investigate the physiological and perceptional responses to different directional modes of shuttle running. The results of this study showed that both lateral and forward-backward shuttle running elicited greater physiological and perceptional responses than forward shuttle running at the selected speed. All variables investigated in this study (HR, %HR_max_, VO_2_, %VO_2max_, [La^–^]_peak_, and RPE) were significantly higher in forward-backward shuttle running than in forward shuttle running and the values in lateral shuttle running were significantly higher than the other two conditions. The findings verified our hypothesis that both lateral and forward-backward shuttle running could produce greater physiological demands compared with forward shuttle running. It indicated that to complete the same distance shuttle running at the same speed, running forwards was more efficient than running backwards or running sideways. The outcome was not surprising. Generally, humans are used to running or walking in the forward direction. Backward and lateral movements are applied in daily activities or sport exercises at times. With the alterations of visual field and neuromuscular activation, running backwards and running laterally are considered as unaccustomed tasks for humans compared with running forwards (Oyeyemi et al., 2017), especially performed continuously. As pointed by Schwane et al. (Schwane et al., 1983; Flynn et al., 1994), to complete a unfamiliar activity may require greater motor unit recruitments, resulting in increased energy cost.

Plenty of evidence has shown that forward shuttle running required higher physiological demands than constant straight-forward running at the same speed (Osgnach et al., 2010; Zago et al., 2018). So far, however, no published study has investigated the physiological responses of shuttle running involved backward and lateral locomotion. Despite all this, several studies concerning the physiological demands and mechanical characteristics about different directional modes of straight running could help us to understand the influence of running mode on the physical responses. In an early study, Reilly and Bowen (1984) determined the energy expenditure and perceptual fatigue of three directional modes (forward, backward, and lateral) of straight running on a treadmill at 5, 7, and 9 km⋅h^–1^. The results showed that both backward running and lateral running produced significantly greater energy demand and higher RPE than forward running at the investigated speeds. In another study (Williford et al., 1998), it was observed that HR and VO_2_ were significantly higher in lateral running and backward running than forward running. These findings were congruent with the results in the present study. However, the results of comparing backward running and lateral running were inconsistent. Reilly and Bowen (1984) reported that neither energy expenditure nor RPE was significantly different between backward and lateral running. Williford et al. (1998) found that the differences between backward running and lateral running were speed dependent. HR and VO_2_ were significantly higher in lateral running at the speed of 3 miles per hour (mph), while no significant difference was found at 5 mph. Besides, differences of the HR and VO_2_ between running modes appeared to dramatically greater at higher speed in these two studies. It indicated that speed and running mode may have an interaction effect on the physiological responses in shuttle running. In our study, lateral shuttle running elicit significantly higher physiological responses than forward-backward mode. Nevertheless, it was difficult to directly compare these findings because forward-backward shuttle running contained forward components. Further researches are needed to determine whether the physiological responses in lateral running is higher than running backwards.

In addition, Mayo et al. (2004) observed that the physiological responses to forward-motion and backward-motion exercise were similar while the lateral-motion exercise elicit significant greater VO_2_ and HR with the similar RER and RPE relative to forward and backward exercise at self-selected intensities. The findings indicated that lateral-motion exercise was performed with higher metabolism at the perceived similar intensities. It should be noted that forward and backward exercise were conducted on a treadmill while lateral-motion exercise was performed on a slideboard. We supposed that the physiological responses to running in different directions were similar at the same perceived intensities. However, the physiological responses to lateral running at the self-selected intensity may need further research.

Forward running and backward running are accomplished in sagittal plane but in opposite direction. In these two kinds of running, the total lower limb muscle work is similar at the same speed, but the muscles are activated concentrically and eccentrically with quite different sequences and extents of activation, accompanied by different ankle, knee, and hip joint moment and power output (Flynn et al., 1994). During forward running, ankle plantar flexors produced more power and played an important role in propulsion, whereas knee extensors were the primary source of propulsion in backward running because of the higher moment and power generation (DeVita and Stribling, 1991). Hip moment and power patterns of forward running and backward running were opposite in direction but similar in magnitude (DeVita and Stribling, 1991). Unlike forward and backward running, lateral running is performed in the frontal plane with prominent hip abduction-adduction and relatively smaller flexion-extension angles of hip, knee, and ankle. Thus, the groups of muscles activated in lateral movement and the magnitude of activation must be distinct from the forward or backward mode. Different neuromuscular firing patterns may be one of the explanations to the distinct physiological and perceptional responses in different modes of shuttle running. The differences of kinetics may be another reason for the different physical responses. Wright and Weyand (2001) investigated the application of ground force during forward running and backward running and noted that metabolic rates during running were associated with the rates of ground force application and the volume of muscle activated to produce support forces against the ground. Unfortunately, few researches have investigated the kinematic parameters and muscle activities of lateral running.

The changes in stride length and stride frequency may also contribute to the different physiological demands. According to the previous studies, subjects employed shorter stride length and higher stride frequency during backward running than during forward running at the same speed. These differences became larger at higher speeds (Flynn et al., 1994; Williford et al., 1998; Wright and Weyand, 2001). During lateral running, stride length was shorter and stride frequency was higher than forward running and backward running (Williford et al., 1998). Changes of stride length and stride frequency in different running modes may be one of reasons that caused distinct physiological and perceptional responses.

In the present study, there were some limitations should be discussed. First, the forward-backward shuttle running mode, other than backward mode, was applied in this study, resulting in the difficulty to compare the effects of backward and lateral modes. Nevertheless, backward shuttle running was difficult to performed and seldom happened in the match. Forward-backward shuttle running was much closer to the practice and often used in sport training. Second, the values at rest were not collected with the assumption of the baseline values of the same subject fluctuated within a narrow range during the test days and the random testing order could offset the variations. In some studies, authors assumed that all participants had the same baseline values, such as 3.5 ml⋅kg^–1^⋅min^–1^ (Buglione and di Prampero, 2013) or 5 ml⋅kg^–1^⋅min^–1^ for resting VO_2_ and 1 mmol⋅L^–1^ for resting [La^–^] (Buglione and di Prampero, 2013; Zamparo et al., 2014, 2015), which were subtracted from the total values to calculate the energy cost during locomotion. Moreover, the large differences of the physiological and perceptional responses between running modes demonstrated that the baseline values could be safely neglected in this study. Therefore, the absence of baseline values had little impact on our conclusions. Third, the present study only examined the physiological and perceptional responses to three modes of shuttle running at the speed of 6 km⋅h^–1^ in 5-m distance. However, shuttle running could be performed at various combinations of speed and distance. Other conditions were not involved in this preliminary study.

## Conclusion

In conclusion, the physiological and perceptional responses in 5-m shuttle running at a mean speed of 6 km⋅h^–1^ depended on the running directional modes, with the responses highest in lateral shuttle running, and lowest in forward shuttle running. When utilized in home-based exercise, shuttle running could stimulate the cardiorespiratory and musculoskeletal system to different extents by performing with different directional modes. This preliminary study only investigated HR, %HR_max_, VO_2_, %VO_2max_, [La^–^]_peak_, and RPE in forward, forward-backward and lateral 5-m shuttle running at 6 km⋅h^–1^. Further researches can be designed to explore more physiological and biomechanical variables at other intensities with various speeds and shuttle distances in order to determine the underlying reasons for these differences.

## Data Availability Statement

The raw data supporting the conclusions of this article will be made available by the authors, without undue reservation.

## Ethics Statement

The studies involving human participants were reviewed and approved by the Research Ethical Committee of Shanghai University of Sport. The patients/participants provided their written informed consent to participate in this study.

## Author Contributions

YL and BL contributed to the study conception. XW, CG, and BL completed the study design. XW, GZ, LH, and MH performed the material preparation and the data collection. CG completed the data analysis and the first draft of the manuscript. YL and GN contributed the critical revision of the manuscript. YL acquired the funding support and facilitated the whole process. All authors have read and approved the final manuscript and agreed to be accountable for all aspects of the work.

## Conflict of Interest

The authors declare that the research was conducted in the absence of any commercial or financial relationships that could be construed as a potential conflict of interest.

## Publisher’s Note

All claims expressed in this article are solely those of the authors and do not necessarily represent those of their affiliated organizations, or those of the publisher, the editors and the reviewers. Any product that may be evaluated in this article, or claim that may be made by its manufacturer, is not guaranteed or endorsed by the publisher.
